# Interpretable Two-Stage Machine Learning for Early and Full Drug Release Prediction in PLGA Microspheres

**DOI:** 10.3390/ph19050767

**Published:** 2026-05-14

**Authors:** Younghun Song, Saroj Bashyal, Hyuk Jun Cho, Mi Ran Woo, Jong Oh Kim, Duhyeong Hwang

**Affiliations:** 1 College of Pharmacy, Keimyung University, Daegu 42601, Republic of Korea; finalpeetbreaker@gmail.com (Y.S.); sarojbashyal63@gmail.com (S.B.); hjcho89@kmu.ac.kr (H.J.C.); 2 Department of Pharmaceutical Engineering, Daegu Catholic University, Gyeongsan 38430, Republic of Korea; mrwoo95@cu.ac.kr; 3 College of Pharmacy, Yeungnam University, Gyeongsan 38541, Republic of Korea; jongohkim@yu.ac.kr

**Keywords:** long-acting injectables, PLGA microspheres, in vitro release, drug release prediction, machine learning

## Abstract

**Background/Objectives:** Poly(lactic-co-glycolic acid) (PLGA) microspheres are widely used in long-acting injectable (LAI) formulations because PLGA exhibits well-established biocompatibility and undergoes controlled hydrolytic degradation into metabolizable byproducts. However, optimization of microspheres typically requires time-consuming in vitro testing. Therefore, we developed a predictive machine learning model for early-stage and full time-dependent release profiles of drug-loaded PLGA microspheres. **Methods:** Using a published dataset comprising 321 release profiles from 89 drugs, we first developed a classification model to identify slow-release behavior (≤20% release within 3 days) and subsequently integrated the predicted early-release probability into a regression model to estimate cumulative release over time. **Results:** Among tree-based ensemble models, XGBoost achieved the lowest mean absolute error (MAE = 0.126) and highest Pearson correlation coefficient (r = 0.831). SHapley Additive exPlanations (SHAP) analysis revealed that drug and polymer molecular weight, predictive slow-release probability, and polymer concentration substantially influence release behavior. We also assessed this framework with external datasets. Drug release data for olaparib-loaded PLGA microspheres were obtained in-house, whereas those for semaglutide-based microspheres were obtained from the published literature. In both datasets, this framework demonstrated low MAE values (0.096 and 0.068, respectively). **Conclusions:** This suggests that the proposed framework can predict in vitro drug release and support efficient optimization of PLGA-based LAI formulations.

## 1. Introduction

Polymeric microspheres have been widely studied for the development of long-acting injectables (LAIs), and several LAI products have received regulatory approval [1,2]. Unlike conventional injectables, LAIs enable sustained drug exposure following a single administration, which can improve patient adherence by reducing dosing frequency [3,4,5]. Among the various polymers employed, poly(lactic-co-glycolic acid) (PLGA) remains a widely used polymer for microspheres due to its biocompatibility and its ability to undergo controlled hydrolytic degradation, enabling sustained drug release [1,6]. However, the development and optimization of polymeric microsphere-based LAIs typically require extensive in vitro release assays to characterize drug release profiles, which are key determinants of pharmacokinetic behavior [7,8,9,10].

Drug release from PLGA-based microspheres is controlled by both diffusion and polymer degradation processes, with their relative contributions depending on the formulation and environmental conditions [11,12]. In the early stage, drug molecules located near the particle surface can rapidly diffuse into the surrounding medium, resulting in a burst release. After that, water penetration into the polymer matrix induces hydrolytic degradation of PLGA, leading to increased porosity. Release behavior may be dominated by diffusion-controlled, degradation-controlled, or a combination of both mechanisms depending on the system [11,12]. More recent work has further underscored the complexity of these processes, emphasizing the role of microparticle internal structure, pore network accessibility, and swelling-induced transitions in release kinetics [13].

To characterize these release behaviors, data are typically collected over 20–30 days and, in some cases, over 150 days [14]. Consequently, formulation optimization generally involves multiple rounds of trial-and-error experimentation, making the process time-consuming [15,16]. Several mathematical models have been introduced to describe drug release behavior from microspheres [12,17]. However, these models are inherently post hoc and rely on experimentally obtained release data for parameter fitting.

Given their clinical utility and regulatory tractability, numerous experimental studies on PLGA-based microspheres have been conducted, resulting in substantial release datasets available [14]. However, effectively leveraging these datasets to capture complex and non-linear release behavior remains challenging. In this context, data-driven approaches, particularly machine learning (ML), have recently emerged as powerful tools for exploring complex design spaces and identifying non-linear relationships in pharmaceutical systems. By learning patterns directly from experimental data, ML models offer a promising strategy for predicting drug release behavior from existing datasets, complementing established mechanistic models that describe diffusion- and degradation-controlled release processes [18,19]. Moreover, ML models provide interpretability by quantifying the contribution of materials, formulation, and process variables [20].

Previous ML models for release prediction have primarily focused on physicochemical properties of the drug and polymer (e.g., molecular weight (MW), topological polar surface area (TPSA), and partition coefficient (logP)) and formulation parameters such as drug loading capacity (DLC), encapsulation efficiency (EE), and particle size [18]. However, such features alone are insufficient to fully capture the complexity of drug release behavior from PLGA-based microspheres [21,22]. For example, even when PLGA MW and the lactic acid-to-glycolic acid ratio (LA/GA ratio) are identical, differences in the end-cap type of PLGA can lead to distinct release profiles [23,24]. Acid-terminated PLGA exhibits faster degradation and accelerated drug release compared to ester-terminated PLGA. In addition, preparation conditions, such as PLGA concentration, influence DLC, EE, and particle size, significantly affecting the release kinetics [25,26,27]. Furthermore, assay conditions, including agitation rate, can directly impact drug release behavior by maintaining the sink condition and enhancing drug diffusion [26,28]. These observations highlight that drug release from PLGA microspheres is governed by multiple interacting factors that are not fully captured by conventional descriptors.

In this work, ML-based predictive models were developed and subsequently interpreted to identify key factors influencing drug release kinetics. Notably, early-stage release behavior—such as burst release or lag release within the first three days—plays a critical role in shaping the subsequent release pattern [22,29]. However, this information is not explicitly incorporated into most existing predictive models. To address this limitation, we developed a data-driven ML framework that integrates both conventional descriptors and early-stage characteristics. Specifically, a classification model was first constructed to predict an early-stage release probability within the first three days, and the predicted probability was subsequently incorporated as an additional feature into a regression model to estimate the full release profile. This stacked classification–regression framework enables improved prediction of time-dependent drug release behavior by capturing both initial release signatures and long-term kinetics.

To implement this approach, a previously published and curated dataset was utilized to ensure data reliability and accessibility [14]. Following model development, interpretation analysis was performed to identify key factors influencing drug release behavior. To evaluate the robustness of the framework, external validation was performed. PLGA microspheres loaded with olaparib (OLA), which was not included in the training dataset, were prepared in-house and used for prospective validation. In addition, external literature data for semaglutide (SGT)-loaded PLGA microspheres were utilized to further assess predictive performance under chemically distinct conditions [30]. Notably, SGT is a peptide drug with physicochemical properties substantially different from those of small-molecule drugs, providing a stringent test of model applicability beyond the primary training distribution [31,32]. Through these validation strategies, the applicability of the model across diverse drug–polymer systems was systematically evaluated.

Our study presents a data-driven framework for predicting drug release from PLGA-based microspheres and provides mechanistic insights into the key factors governing release kinetics. This approach offers a scalable strategy for accelerating formulation design and reducing reliance on time-consuming experimental screening.

## 2. Results

### 2.1. Early-Stage Classification

A total of 314 formulation indices were used for slow-release probability prediction (Figure S1). Among them, 132 formulations were labeled as slow-release (indicator = 1), and 182 formulations were labeled as non-slow-release (indicator = 0).

**Figure 1:**
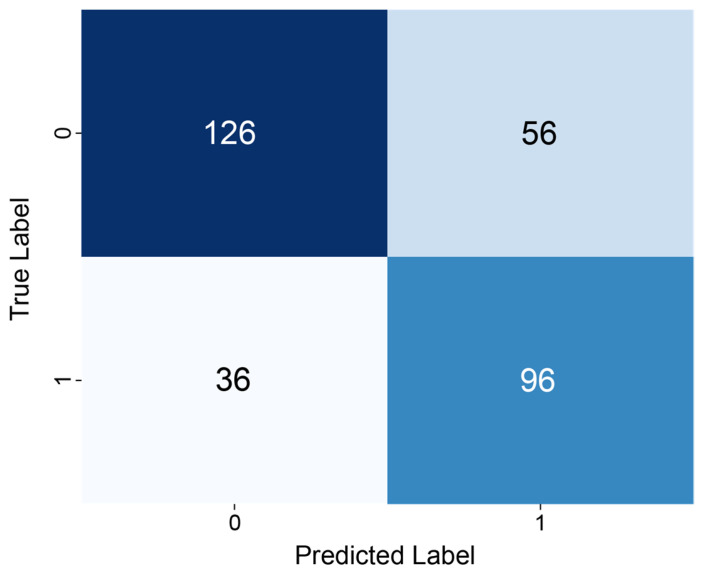
Confusion matrix for slow-release probability prediction. The confusion matrix summarizes the performance of the logistic regression model based on OOF predictions generated using a Group *k*-fold splitting strategy (*k* = 10). The model identified 96 slow-release formulations correctly (true positives) and 126 non-slow-release formulations (true negatives). However, the model produced 36 false negatives and 56 false positives. The threshold of 0.5 was applied to convert predicted values into binary labels.

The aggregated confusion matrix based on OOF predictions generated using a 10-fold group-based splitting strategy is shown in Figure 1. Among the 132 true slow-release formulations, 96 were correctly classified, while 36 were misclassified as non-slow-release. Among the 182 non-slow-release formulations, 126 were correctly predicted and 56 were misclassified as slow-release. The overall classification performance metrics are summarized in Table 1. The model achieved an accuracy of 0.707, a recall of 0.727, a precision of 0.632, and an F1-score of 0.676.

### 2.2. Feature Correlation Analysis

**Figure 2:**
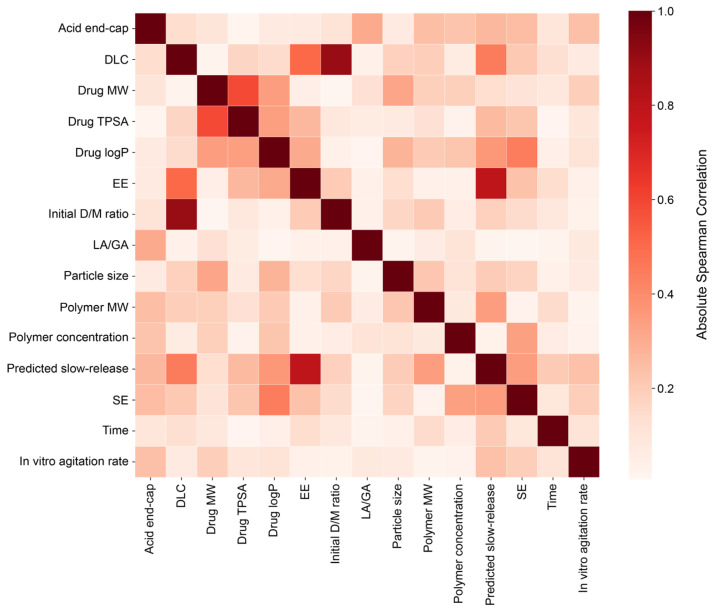
Heatmap of the absolute Spearman’s rank correlation coefficients among input features used for regression modeling. Absolute Spearman’s rank correlation coefficients were calculated to evaluate pairwise monotonic relationships between formulation, physicochemical, and process-related features.

Absolute Spearman’s rank correlation coefficients (ρ) were calculated for relationships among input features in the full release prediction model. The results are summarized in Figure 2. Most feature pairs showed low absolute correlation coefficients below 0.5. This indicates that these features do not exhibit strong monotonic associations. However, several feature combinations exhibited high correlation coefficients above 0.5. DLC and initial D/M ratio showed the highest correlation in the heatmap (ρ = 0.902). EE and the predicted slow-release probability showed a secondary rank correlation (ρ = 0.793). MW and TPSA of the drug (ρ = 0.589) and EE and DLC (ρ = 0.506) also demonstrated moderate correlations.

### 2.3. Total Release Regression

A total of 4820 release data points were used for time-dependent regression modeling of drug-loaded PLGA microspheres. Performance evaluation was conducted for each regression algorithm using Group *k*-fold cross-validation (*k* = 5). Detailed fold-wise results are provided in Table S1, and the mean performance across folds for each model is summarized in Table 2.

Among the evaluated models, XGB demonstrated the best overall performance, achieving a mean MAE of 0.126 (±0.011). RF and LGBM also showed competitive performance, with mean MAE values of 0.133 (±0.010) and 0.135 (±0.015), respectively. The best-performing fold of the XGB model achieved an MAE of 0.113 and a Pearson r of 0.867. The hyperparameters of each regression model are summarized in Table S2.

The additional internal validation results of the final selected XGB are summarized in Table 3. Under repeated group-based resampling, the model achieved a mean MAE of 0.123 (±0.014) and a Pearson r of 0.836 (±0.041), indicating consistent performance across different group assignments. In contrast, LODO validation resulted in a higher MAE of 0.174 (±0.075) and a Pearson r of 0.833 (±0.121), reflecting increased variability across drugs. All results of validation are summarized in Tables S3 and S4.

### 2.4. SHAP Analysis

**Figure 3:**
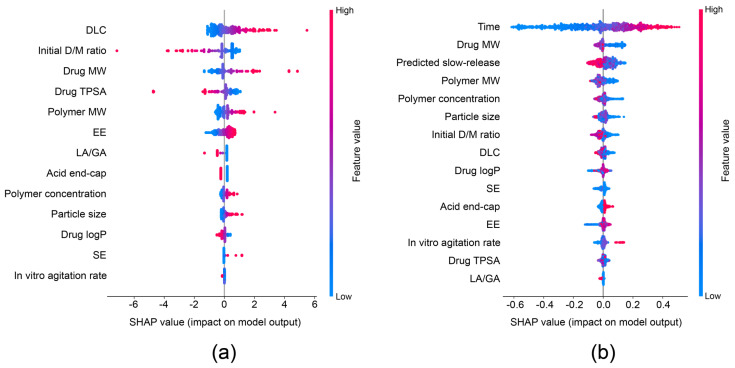
SHAP analysis of classification model and regression model: (**a**) SHAP analysis of logistic regression model for slow-release probability prediction; (**b**) SHAP analysis of XGB regression model.

SHAP analysis was performed to quantify the contribution of individual features to the model predictions (Figure 3). For the slow-release classification model (Figure 3a), DLC exhibited the largest impact on model output, followed by initial D/M ratio and drug MW. Higher DLC values were associated with positive SHAP values, indicating a stronger contribution toward slow-release prediction. The MW of the drug and polymer also showed a considerable positive influence on model output. In contrast, initial D/M ratio and drug TPSA showed a negative contribution to slow-release prediction. However, in vitro agitation rate demonstrated comparatively smaller SHAP magnitudes.

For the full time-dependent fractional release prediction model, time showed the highest SHAP impact as expected (Figure 3b). Drug MW and predicted slow-release probabilities showed noticeable contributions to model predictions following time. MW descriptors and predicted probabilities showed negative effects on fractional release. Moreover, polymer concentration showed large SHAP values and negatively contributed to the model output. In contrast, acid end-cap and in vitro agitation rate showed positive contributions, although their SHAP magnitudes were relatively small.

### 2.5. External Validation

**Figure 4:**
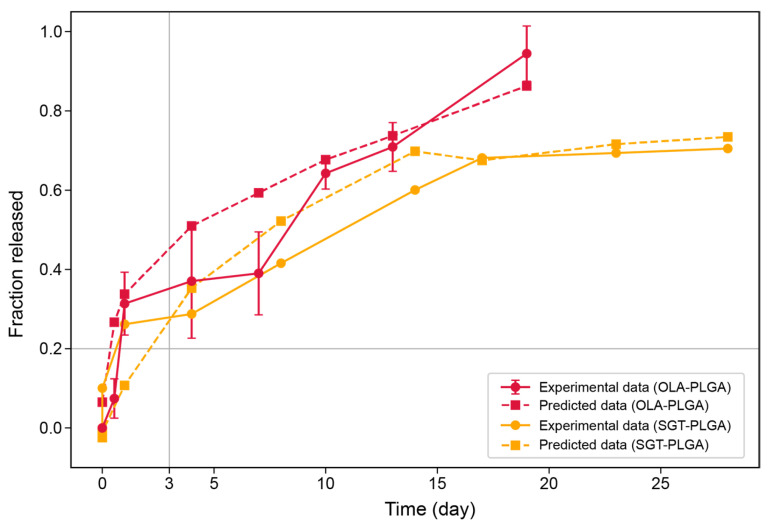
Predicted and experimental in vitro fractional drug release profiles for OLA-PLGA microspheres and SGT-PLGA microspheres used for external validation. Error bars represent standard deviation (*n* = 3).

External validation was performed using two independent drug–polymer systems, SGT-PLGA microspheres and OLA-PLGA microspheres. The predictive performance is summarized in Table 4, and the predicted and experimental release profiles are shown in Figure 4. For SGT-PLGA microspheres, the slow-release probability prediction model yielded values greater than 0.999 and the regression model achieved an MAE of 0.068 and a Pearson correlation coefficient of 0.971. For OLA-PLGA microspheres, the predicted slow-release probability was 0.082 using the classification model and the MAE was 0.096 and Pearson r was 0.962 using the regression model.

As shown in Figure 4, the predicted release curves followed the experimental release profiles across the evaluated time range for both formulations. The predicted values closely matched the experimental fractional release at early, intermediate, and late time points. Although the predicted slow-release probability of SGT-PLGA microspheres was close to 1, the predicted release value exceeded a fraction of 0.2 within the three days. Moreover, deviations between predicted and experimental values were observed between days 2 and 10 for the OLA-PLGA formulation.

## 3. Discussion

In this study, we developed a two-stage ML framework for the in vitro release prediction of PLGA microspheres. A classification model was used to characterize the early phase release. The slow-release indicator was defined based on a release fraction of 0.2 within the first three days and used as the target for the first model. After that, a regression model was applied to describe the full time-dependent release behavior over the full duration. The framework showed excellent performance on internal datasets and was evaluated using external datasets to examine robustness under limited external conditions. SHAP analysis provided interpretable insights into the important features affecting in vitro release. Overall, total in vitro release patterns can be estimated from physicochemical properties and formulation parameters. It provides a data-driven approach that can guide formulation design and help reduce the extent of trial-and-error experimentation, while remaining grounded in experimentally derived data.

First, we employed logistic regression for the slow-release probability classification. This model is less prone to overfitting when applied to small datasets. A total of 314 formulation indices were assigned binary labels based on whether the release fraction exceeded 0.2 within the first three days. This resulted in a balanced distribution of target classes, with 132 formulations labeled as slow-release and 182 as fast-release. Such balance avoided class bias during training and contributed to the predictive performance. As a result, the model yielded evaluation metrics of approximately 0.7 for accuracy, recall, and F1-score. Despite the relatively small dataset, the predicted slow-release probability served as an informative feature [33].

For the full release prediction, XGB was chosen among the regression models. Moreover, other ensemble methods (RF and LGBM) also showed strong predictive performance compared with other models. This reflects their ability to characterize non-linear relationships and interaction effects of various features. Additional internal validation analyses supported the robustness of XGB. Under repeated group-based resampling, the model showed consistent performance across different data splits. In contrast, LODO validation resulted in reduced performance, reflecting the greater difficulty of predicting release profiles for entirely unseen drugs. Despite this, the model retained reasonable predictive capability, suggesting that it can estimate generalizable patterns affecting drug release beyond specific drug–polymer combinations.

To examine relationships among input features, Spearman’s correlation analysis was performed. Most feature pairs showed weak associations and had minimal redundancy. However, four pairs exceeded |ρ| > 0.5: DLC and initial D/M ratio, EE and the predicted slow-release probability, the MW and TPSA of the drug, and EE and DLC. The strong correlation between DLC and D/M ratio likely arises from their mathematical dependency, as DLC is derived from the initial D/M ratio. The association between EE and the predicted slow-release probability may reflect formulation characteristics such as matrix stability and drug encapsulation, which are known to affect release behavior [23,34]. The relationship between drug MW and TPSA is considered as an intrinsic molecular property. Both descriptors increase with molecular complexity and may influence diffusion in the same way.

SHAP analysis was performed to interpret feature importances in both the slow-release classification and total release regression models. In the slow-release classification model, DLC, initial D/M ratio, drug MW, drug TPSA, and polymer MW were identified as the most influential features. A higher DLC, drug MW, and polymer MW were associated with increased probabilities of slow release within three days. This is consistent with the reduced diffusivity of larger drug molecules and slower polymer degradation kinetics of higher-molecular-weight PLGA, which can delay water penetration and limit the formulation of continuous diffusion pathways within the polymer matrix [15,23]. In contrast, a higher TPSA and higher initial D/M ratio were associated with faster release in the early phase. Increased polarity may promote aqueous partitioning over polymer retention. Furthermore, higher initial drug amounts have been linked to increased drug fractions near the surface of microspheres, which can facilitate early pore network access to the release medium and contribute to burst release behavior [13,26].

In the full time-dependent release regression model, time was the dominant contributor owing to its intrinsic role in cumulative release progression [17,18]. Drug MW, the predicted slow-release probability, polymer MW, polymer concentration, and particle size also showed a significant influence on the model. Larger drug and polymer molecular weights were generally associated with slower release. This is likely due to slower diffusion and polymer degradation, which governs PLGA erosion through molecular weight-dependent degradation kinetics and oligomer formulation [11,15,23,35,36]. In addition, the time-dependent evolution of drug release reflects the transition from diffusion-dominated transport in the early stages to degradation- and swelling-controlled mechanisms at later stages [13]. This transition is consistent with established mechanistic models, where early drug release is governed by diffusion of surface-associated drugs, while the later phase is dominated by polymer degradation and erosion processes [12]. The slow-release predictive probabilities showed negative contributions to drug release due to their intrinsic role. Nevertheless, their importance remained lower than that of time and drug MW. This suggests that they do not govern the overall release behavior and can be attributed mainly to early-stage release, while the later stages are increasingly influenced by structural evolution of the microparticles, such as pore network accessibility and swelling-induced changes in polymer permeability.

Among the additional formulation features, acid end-cap and polymer concentration consistently contributed to both models. Acid-terminated PLGA was associated with faster release. This trait accelerated hydrolytic degradation and autocatalytic effects [23,24]. However, higher polymer concentration was associated with slower release. It reflected increased matrix density and reduced diffusion pathways [25,26,27]. In vitro agitation rate demonstrated a clear positive contribution in the regression model under stronger mixing conditions [26,28].

Prospectively prepared in-house data was used for the external evaluation of the framework. OLA shares similar physicochemical properties with the drug in the training dataset. OLA-loaded PLGA microspheres were therefore selected for validation. Predicted results showed similar trends to experimental data. Some discrepancies emerged between days 7 and 10. This interval showed the highest variability and standard deviation in the experimental data. This may reflect a transient acceleration in drug release in the mid-phase. Such abrupt changes are challenging to capture when the model is mainly trained on smoother release trajectories. Nevertheless, the proposed framework is useful for predicting drug release patterns.

Next, we evaluated the proposed framework using SGT-loaded PLGA microspheres from an independent literature dataset [30]. SGT has physicochemical properties distinct from those of the training dataset, particularly its relatively high molecular weight, providing a stringent test case [10]. The predicted release profiles closely matched the experimental trends across the full time course. However, a deviation was observed at early time points: the predicted slow-release probability was close to 1, whereas the regression model predicted release values exceeding 20% within 3 days. This arises because the slow-release indicator is incorporated as a probabilistic prior rather than a strict constraint, and the final prediction reflects the combined influence of multiple features. As the SGT system lies outside the primary training distribution, other dominant features can modulate the prediction beyond the indicator. Despite this mismatch, the slow-release probability contributed to an overall tendency toward slower predicted release, indicating that it remains informative. These results suggest that the model can capture overall release behavior even for chemically distinct, higher-molecular-weight compounds, while the reduced accuracy in early-stage prediction highlights the need to include more high-molecular-weight drug-loaded microsphere datasets in future training.

Despite a strong performance on both internal and external datasets, the model was developed based on a single published dataset, and feature importance patterns may vary depending on dataset composition [21]. Therefore, validation using additional well-structured curated datasets is warranted to confirm the robustness of the models. Moreover, abrupt or pronounced burst release behaviors were relatively underrepresented in the training data, which may limit the ability of the model to capture non-smooth release transitions. A notable point of the current framework is that dissolution medium properties such as pH, ionic strength, and surfactant content are not encoded as input features. Because the training data were dominated by parenteral LAI profiles acquired in PBS at a physiological pH, the model is applicable to predictions under similar conditions. For ionizable drugs whose solubility and partitioning depend strongly on a medium pH, predictions outside this physiological window should be interpreted with caution. Such conditions lie beyond the current scope of the framework but represent a meaningful consideration for future model extensions to dissolution environments with a substantially different pH, such as oral or biorelevant release conditions.

## 4. Materials and Methods

### 4.1. Data Description

A previously published dataset of drug-loaded PLGA microspheres comprising 321 release profiles from 89 drugs was used for model development [14]. Each release profile contained approximately 10–15 time points, resulting in a total of 4913 individual release data points. Only release profiles spanning at least 72 h and reporting a final cumulative release exceeding 60% were included in the dataset. All included release profiles were acquired predominantly in phosphate-buffered saline (PBS) at a physiologically relevant pH of 7.4 and 37 °C consistent with the standard in vitro testing condition for parenteral PLGA-based long-acting injectable formulations [14].

This dataset includes various features related to drug and polymer properties, formulation parameters, and the in vitro condition. Feature names used in the model are provided in parentheses. Drug properties included MW of the drug (Drug MW), TPSA of the drug (Drug TPSA), and logP of the drug (Drug logP). PLGA properties included MW of the PLGA (Polymer MW) and LA/GA ratio (LA/GA). Formulation parameters included the initial drug-to-polymer ratio (Initial D/M ratio), particle size of the formulated microspheres (Particle size), drug loading capacity which was expressed as the mass percentage of drug encompassed in the microspheres (DLC), and drug encapsulation efficiency representing the ratio of encapsulated drug mass relative to the initially used drug mass (EE). Moreover, the concentration of solubility enhancer in the in vitro release medium (SE) was included as an in vitro condition. Time, expressed in days, was also included as a continuous variable for regression modeling. Additional formulation and process-related features were engineered as described below.

### 4.2. Feature Engineering

The features originally provided in the published dataset, along with the additional engineered features introduced in this study, are summarized in Table 5. To construct an early-stage release indicator, release and time data were examined for each formulation. Formulations exhibiting ≤20% cumulative release within the first three days were defined as slow-release systems. This criterion was encoded as a binary variable (Slow-release indicator) and used as the target variable for the classification model. Additional features were incorporated based on formulation details extracted from the corresponding source literature for each formulation index. If the required information for a given feature was unavailable in the source publication, the corresponding formulation index was excluded from the dataset to ensure data consistency. All preprocessing procedures were performed in Python (version 3.12.13) using Pandas (version 2.2.2).

In addition, the agitation rate used during the in vitro release assay was incorporated (In vitro agitation rate). The concentration of PLGA in the organic phase was defined as the weight fraction of polymer relative to the total mass of the organic phase (Polymer concentration). Finally, the polymer end-group type was encoded as a binary variable, where acid-terminated PLGA was assigned a value of 1 and ester-terminated PLGA was assigned a value of 0 (Acid end-cap).

### 4.3. ML Model Development

**Figure 5:**
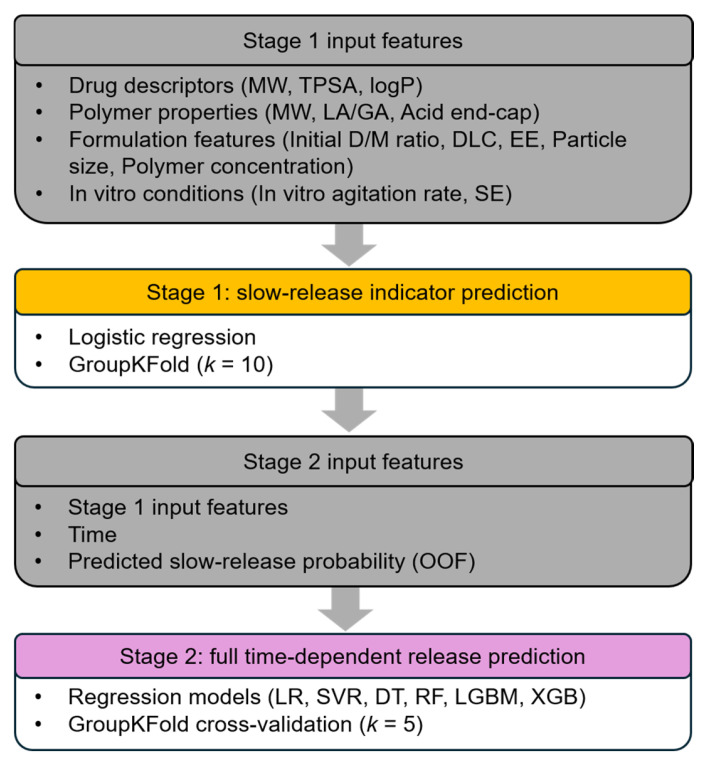
Two-stage machine learning framework to predict fractional release of drug-loaded PLGA microspheres.

A two-stage ML framework was developed to predict drug release from PLGA microspheres (Figure 5). The input features consisted of published dataset features and additional engineered formulation and process-related features.

In stage 1, a classification model was developed to predict the slow-release probability. All input features except time were used as predictors. To prevent data leakage, data splitting was performed using Group *k*-fold based on the formulation index, ensuring that all release points from the same formulation were assigned exclusively to either the training or testing set. A Group *k*-fold strategy was applied (*k* = 10), where logistic regression was trained on nine folds and evaluated on the held-out group. Logistic regression with L2 regularization was used as the classification model, with a regularization strength of 0.5 and the “lbfgs” solver. This procedure was repeated across all folds to generate predicted slow-release probability (Predicted slow-release) for the entire dataset. Out-of-fold (OOF) predictions from stage 1 were used to construct the slow-release feature. These predicted probabilities were subsequently used as an additional input feature in the stage 2 regression model.

In stage 2, a regression model was developed to predict the full time-dependent release profile. The input variables included the original dataset features, additional engineered features, the predicted slow-release probability from stage 1, and time. Regression modeling was performed using Group *k*-fold cross-validation (*k* = 5). Multiple algorithms were evaluated, including linear regression (LR), support vector regression (SVR), decision tree (DT), random forest (RF), extreme gradient boosting (XGBoost, XGB), and light gradient boosting machine (LightGBM, LGBM) [37,38,39]. Machine learning models were implemented using Scikit-learn (version 1.6.1), NumPy (version 2.0.2), XGBoost (version 3.2.0), and LightGBM (version 4.6.0). Hyperparameters were predefined and applied within each training fold, and the best-performing model was selected based on cross-validation performance.

### 4.4. Internal Validation

For further assessment of the robustness of the final selected model, two additional internal validation analyses were performed. First, uncertainty analysis was conducted using repeated group-based resampling with GroupShuffleSplit, in which the assignment of formulation indices to training and test sets was randomly reassigned across 30 repetitions while preserving group integrity. In addition, leave-one-drug-out (LODO) cross-validation was performed to evaluate the model’s ability to predict the release of unseen drug-loaded microspheres. In each iteration, all release profiles corresponding to a single drug were excluded from the training set and used as the test set. This procedure was repeated for all drugs in the dataset, ensuring that no information from the same drug was shared between training and test sets.

### 4.5. Metrics

Two-stage ML models were assessed using different metrics. Classification performance was evaluated using accuracy, recall, precision, and F1-score in Equations (1)–(4). Predicted probabilities were converted into binary labels using a threshold of 0.5 to compute the confusion matrix and associated metrics. Accuracy represents the proportion of correctly classified samples, while recall quantifies the model’s ability to correctly identify slow-release formulations. Precision measures the proportion of correctly predicted positive samples among all predicted positives. F1-score was calculated as the harmonic mean of precision and recall.

(1) $$\mathrm{Accuracy}=\frac{TP+TN}{TP+TN+FP+FN}$$

(2) $$\mathrm{Recall}=\frac{TP}{TP+FN}$$

(3) $$\mathrm{Precision}=\frac{TP}{TP+FP}$$

(4) $$F1\text{-}\mathrm{score}=2\times \frac{Precision\times Recall}{Precision+Recall}$$

where TP, TN, FP, and FN denote true positives, true negatives, false positives, and false negatives, respectively.

Regression performance was assessed using mean absolute error (MAE) and Pearson correlation coefficient (r). MAE measures the average absolute deviation between predicted and experimental release values in Equation (5) and Pearson r evaluates the linear agreement between predicted and actual release profiles.

(5) $$\mathrm{MAE}=\frac{\sum_{i=1}^{n}|\hat{y_{i}}-y_{i}|}{n}$$

where $\hat{y_{i}}$ is the predicted release fraction; $y_{i}$ is the experimental release fraction; *n* is the total number of data points.

### 4.6. Model Interpretation

SHapley Additive exPlanations (SHAP) analysis was conducted to evaluate feature importance and interpret the contribution of input variables in both the early-stage release classification model and the full release profile regression model. For the slow-release classification model, SHAP analysis was performed on the model trained using the entire formulation dataset. For the full release profile prediction model, SHAP values were calculated based on the best-performing fold of the selected optimal ML model. This approach enabled assessment of the relative influence of input features on model predictions. Feature contributions were visualized using force plots generated with the SHAP library (version 0.51.0) in Python (version 3.12.13) [20,40].

### 4.7. Statistical Analysis

To evaluate potential multicollinearity and monotonic relationships among input features, Spearman’s rank correlation coefficients (ρ) were calculated for all feature pairs. Absolute correlation coefficients were computed to identify the associations of input features. Spearman’s correlation was selected due to its robustness to non-linear relationships. The correlation matrix was visualized using a heatmap to identify associated feature pairs prior to second stage model training.

### 4.8. SGT-Loaded PLGA Microspheres

The SGT-loaded PLGA microsphere dataset was from the previously published literature [30]. There are various depots with different formulation conditions. Among them, depot 6 in this literature was selected for external validation in this study. Resomer^®^ RG 504 H (LA:GA = 50:50; MW = 38–54 kDa; acid-terminated) was used for depot 6 and any additives (e.g., methionine or D-mannitol) were not included in the in vitro assay. Compared with other depots reported in the literature, depot 6 showed uniform particle size distribution (D50 ≈ 27 μm, span < 1.35), high EE (~98%), and moderate drug loading (~10%). By selecting depot 6, external validation was conducted to assess the performance of the proposed model under distinct physicochemical conditions.

### 4.9. OLA-Loaded PLGA Microspheres

#### 4.9.1. Materials

Resomer^®^ RG 503 H (PLGA; LA:GA = 50:50; MW = 24–38 kDa), poly(vinyl alcohol) (PVA) (MW = 9–10 k, 80% hydrolyzed), sodium dodecyl sulfate (SDS), acetonitrile (ACN), dimethyl sulfoxide (DMSO), dichloromethane (DCM, HPLC-grade), methanol (HPLC-grade), and phosphate-buffered saline (PBS, pH 7.2) were purchased from Sigma-Aldrich (St. Louis, MO, USA). OLA was purchased from Aladdin Scientific (Shanghai, China).

#### 4.9.2. Formulation Preparation

PLGA microspheres were prepared using an oil-in-water (o/w) single emulsion solvent evaporation method. Briefly, 80 mg of PLGA and 20 mg of OLA were dissolved in 1.6 mL of a solvent mixture (DCM:DMSO = 1:1, *v*/*v*) in a 20 mL scintillation vial to form the organic phase. The organic phase was subsequently poured into 53 mL of DI water containing 0.5 wt% PVA, which had been previously cooled to 4 °C, and homogenized at 2000 rpm for 30 min to generate an oil-in-water emulsion. Following homogenization, the emulsion was transferred into 133 mL of pre-cooled DI water (without PVA) and stirred at 500 rpm for 1 h to facilitate solvent evaporation and microsphere solidification. The resulting suspension was passed sequentially through 100 µm and 70 µm sterile cell strainers to collect microspheres within the target size range. The retained microspheres were collected and lyophilized overnight to obtain dry OLA-loaded PLGA microspheres.

#### 4.9.3. Drug Loading Assay

Drug loading was determined by weighing approximately 2 mg of lyophilized microspheres. A total of 1 mL of ACN was used for extraction of the encapsulated drug. The mixture was vortexed until the microspheres were completely dissolved. The solution was filtered through a 0.2 µm syringe filter and diluted with ACN prior to analysis. Drug quantification was performed using high-performance liquid chromatography (HPLC) on an Agilent 1260 Infinity II system equipped with a photodiode array detector and an XDB-C18 column (4.6 × 150 mm, ZORBAX Eclipse, Agilent Technologies, Santa Clara, CA, USA). The mobile phase consisted of a mixture of DI water and ACN (65:35, *w*/*w*) containing 0.1 wt% formic acid and the flow rate of the mobile phase was 1 mL/min. Detection was carried out at a wavelength of 254 nm.

#### 4.9.4. In Vitro Release Assay

To characterize the in vitro drug release profile of OLA-loaded PLGA microspheres, approximately 2 mg of OLA microspheres was placed in 3.5 kDa dialysis tubes. The dialysis tubes were immersed in release medium consisting of PBS containing 0.5% SDS. The release study was conducted at 37 °C under continuous magnetic stirring at 100 rpm. At predetermined time points (0.5, 1, 4, 7, 10, 13, and 19 days), three independent samples were collected for analysis (*n* = 3). At each sampling time point, the dialysis tubes corresponding to each time point were withdrawn from the release medium, and the amount of remaining drug was quantified. Drug analysis was performed using HPLC under the same conditions used for the drug loading assay. The fractional drug release was calculated by subtracting the amount of drug remaining in the dialysis tube from the initial amount of drug loaded in the microspheres.

## 5. Conclusions

In this study, a two-stage ML framework was developed to predict the release profiles of PLGA microspheres and to analyze the contributions of features. The framework captured release behavior by integrating the predicted early-stage release probabilities with physicochemical and formulation factors. It demonstrated strong predictive performance using an internal dataset and showed robustness in limited external validation cases. These findings suggest its potential utility in supporting the design of PLGA-based formulations under conditions similar to those evaluated in this study, while reducing reliance on time-intensive in vitro testing.

## Figures and Tables

**Table 1:** Classification performance metrics for slow-release probability prediction using logistic regression. Performance metrics were calculated based on OOF predictions. Accuracy, precision, recall, and F1-score were computed by comparing predicted binary labels (threshold = 0.5) with true slow-release indicators.

Accuracy	Precision	Recall	F1-Score
0.707	0.632	0.727	0.676

**Table 2:** Performance comparison of regression models for total release prediction. MAE and Pearson correlation coefficient (r) are reported as mean (standard deviation) across Group *k*-fold cross-validation (*k* = 5). Detailed fold-wise results are provided in Table S1.

ML Model	MAE	Pearson r
LR	0.254 (0.014)	0.475 (0.037)
SVR	0.194 (0.018)	0.684 (0.044)
DT	0.155 (0.008)	0.753 (0.035)
RF	0.133 (0.010)	0.833 (0.037)
LGBM	0.135 (0.015)	0.814 (0.058)
XGB	0.126 (0.011)	0.831 (0.036)

**Table 3:** Internal validation of XGB using uncertainty analysis and leave-one-drug-out (LODO) cross-validation. MAE and Pearson correlation coefficient (r) are presented as mean (standard deviation). All results are provided in Tables S3 and S4.

Method	MAE	Pearson r
Uncertainty analysis	0.123 (0.014)	0.836 (0.041)
LODO	0.174 (0.075)	0.833 (0.121)

**Table 4:** Results of performance metrics for external validation using SGT-PLGA and OLA-PLGA microsphere dataset. MAE and Pearson correlation coefficient (r) were calculated between predicted and experimental release values.

Drug-Loaded Microsphere	Predicted Slow-Release	MAE	Pearson r
SGT-PLGA	>0.999	0.068	0.971
OLA-PLGA	0.082	0.096	0.962

**Table 5:** All the features used in this study.

Criteria	Features
All features from published dataset	Polymer MW
	LA/GA
	Initial D/M ratio
	Drug MW
	Drug TPSA
	Drug logP
	Particle size
	DLC
	EE
	SE
	Time
Preprocessed target from release	Slow-release indicator
Additional features from the literature	In vitro agitation rate
	Polymer concentration
	Acid end-cap

## Data Availability

The datasets used in this paper are publicly available; additional or processed datasets generated and analyzed during the current study are available from the corresponding author upon reasonable request.

## Supplementary Materials

The following supporting information can be downloaded at: https://www.mdpi.com/article/10.3390/ph19050767/s1, Supplementary Materials S1: Figure S1: Workflow of dataset preprocessing and exclusion criteria for model development; Table S2: Hyperparameters of the regression models. Supplementary Materials S2: Table S1: Cross-validation results across all folds for different regression models in full time-dependent release prediction; Table S3: Uncertainty analysis of the XGB model using repeated group-based resampling; Table S4: Leave-one-drug-out (LODO) validation results of the XGB model.

## Abbreviations

The following abbreviations are used in this manuscript:

ACN	Acetonitrile
DCM	Dichloromethane
DLC	Drug loading capacity
DMSO	Dimethylsulfoxide
DT	Decision tree
EE	Encapsulation efficiency
HPLC	High-performance liquid chromatography
LA/GA	Lactic acid/glycolic acid
LAI	Long-acting injectable
LGBM	Light gradient boosting machine
LODO	Leave-one-drug-out
LR	Linear regression
MAE	Mean absolute error
ML	Machine learning
MW	Molecular weight
OLA	Olaparib
OOF	Out-of-fold
PBS	Phosphate-buffered saline
PLGA	Poly(lactide-co-glycolic acid)
PVA	Poly(vinyl alcohol)
RF	Random forest
SDS	Sodium dodecyl sulfate
SGT	Semaglutide
SHAP	Sharpley additive explanation
SVR	Support vector regression
TPSA	Topological polar surface area
