# Supplementary material for: Interpretable Two-Stage Machine Learning for Early and Full Drug Release Prediction in PLGA Microspheres
Source: Pharmaceuticals (Basel). 2026 May 14;19(5):767. doi: 10.3390/ph19050767 (PMC13209779; doi:10.3390/ph19050767)
Supplement: Supplementary file 1 [file pharmaceuticals-19-00767-s001.zip › Supplementary materials S1.pdf]

Supplementary Materials: Figure S1 and Table S2

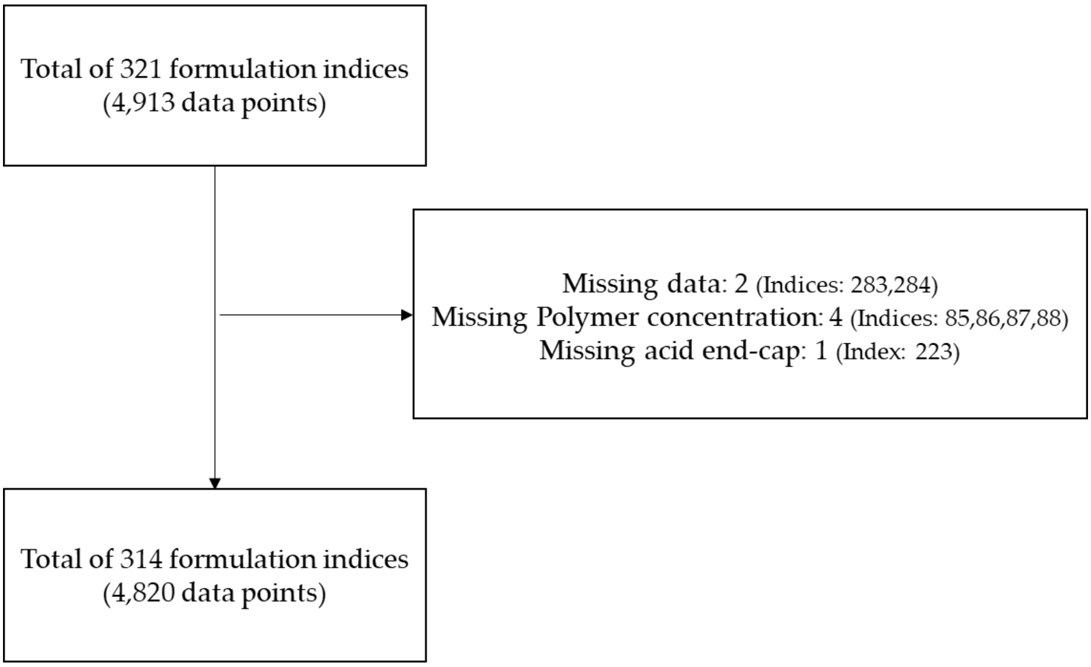

Figure S1. Workflow of dataset preprocessing and exclusion criteria for model development.

Table S2. Hyperparameters of the regression models.

Model	Hyperparameters
LR	None
SVR	kernel = RBF; C = 10; epsilon = 0.05
DT	max_depth = 10; min_samples_split = 5; min_samples_leaf = 2
RF	n_estimators = 600; max_depth = 15; min_samples_split = 5; min_samples_leaf = 2; max_features = sqrt
LGBM	n_estimators = 500; learning_rate = 0.1; num_leaves = 16; min_child_samples = 40; subsample = 0.6
XGB	n_estimators = 600; learning_rate = 0.05; max_depth = 15; subsample = 0.7; colsample_bytree = 0.7

Note: LR, linear regression; SVR, support vector machine; DT, decision tree; RF, random forest; LGBM, light gradient boosting machine; XGB, extreme gradient boosting.
